# Preference for initiation of end-of-life care discussion in Indonesia: a quantitative study

**DOI:** 10.1186/s12904-021-00894-0

**Published:** 2022-01-06

**Authors:** Venita Eng, Victoria Hewitt, Aria Kekalih

**Affiliations:** 1 Indonesian Cancer Foundation Jakarta Chapter, Jalan Baru Sunter Permai Raya no.2, Jakarta Utara, Jakarta, 14340 Indonesia; 2 Newcastle University, Newcastle Upon Tyne, NE1 7RU UK; 3 Master Program in Occupational Medicine, Department of Community Medicine, Universitas Indonesia, Jl. Pegangsaan Timur No.16, RT.1/RW.1, Pegangsaan, Kec. Menteng, Kota Jakarta Pusat, Jakarta, 10310 Indonesia

**Keywords:** End-of-life care, Communication, Preference, Developing countries, Online questionnaire

## Abstract

**Background:**

Initiating discussion about death and dying is often considered a difficult topic for healthcare providers, thus there is a need for further research to understand this area, particularly in developing countries. The aim of this study was to describe preferences for the initiation of end-of-life care discussions in Indonesia, comparing the general population and health care professionals.

**Methods:**

This cross-sectional, descriptive study analysed quantitative data from 368 respondents to an online questionnaire (255 general population (69%); 113 healthcare professionals (31%)) utilizing consecutive sampling and snowball sampling methods.

**Results:**

Overall, most respondents (80%) stated that they would like to discuss end-of-life issues with a healthcare professional in the case of terminal illness. This was more marked amongst healthcare professionals compared with the general population (94% vs. 75%, respectively, *p* < 0,001). The preferred time for discussion was at first diagnosis (68% general population, 52% healthcare professionals, *p* = 0.017) and the preferred person to start the discussion was the doctor (59% general population, 71% healthcare professionals, *p* = 0.036). Fewer respondents wanted to know about prognosis compared to diagnosis (overall 76% v 93% respectively).

**Conclusion:**

Doctors have vital role in end-of-life care discussion, and attempts should be made to encourage physicians to initiate these conversations and respond to patient’s requests when needed. These findings contribute to the existing body of knowledge in this area of practice, with focus on a developing country. The role of socio-cultural influences on these conversations warrants further research, in order to develop practical resources to support clinicians to appropriately conduct end-of-life care discussions with their patients and to provide data for policymakers to develop services.

**Supplementary Information:**

The online version contains supplementary material available at 10.1186/s12904-021-00894-0.

## Introduction

### Background

It is widely acknowledged that the ability of patients to express their personal preferences, especially regarding end-of-life care, is a determinant of healthcare quality [1, 2]. End-of-life care applies to patients with advanced incurable disease in which death is expected within 12 months and consists of the palliative approach to enable living as actively as possible and dying with dignity [3]. End-of-life discussions comprise options for treatment, place of care, healthcare personnel involvement, and other choices to ensure comfort and good quality of life [2].

### Barriers to discussing end of life care

The UK’s National Institute for Health and Care Excellence recommends that care at the end of life should align to the patient’s needs and choices, situated as much as possible in their preferred place [1]. As a patient deteriorates, their ability to make independent and autonomous decisions may decline and the opportunity to fully consider and express preferences regarding their care is lost. Discussions about end-of-life care should, therefore, begin where possible in early stage of the disease [4] yet many patients do not communicate their preferences [5] due a range of reasons including lack of mental capacity [4], failure of healthcare professionals to initiate discussion [5] and lack of resource and education [6]. Clinician-related factors presenting barriers to discussing end-of-life with patients encompass lack of experience, lack of resources including time, cultural differences, difficulty in prognostication and perceived reluctance of patient or family member [6]. Fear of causing emotional harm to the patient often results in discussions beginning late in the progression of disease [4].

### Developing countries

The need to further explore end-of-life care preferences as a basis for practice recommendations and guidelines is acknowledged [7]. Most research in this area is situated in developed economies, despite the greatest burden of advanced disease being borne by low and middle income countries [8].

This study was conducted in Indonesia, a lower middle income country with a population of 274 million and median age of 29.7 years [9]. The majority of the population (62%) has attained education below level 3 of the International Standard Classification of Education [10] (upper secondary) with 12% completing tertiary education at levels 5 (undergraduate) through 8 (doctorate) [11]. In common with other developing countries, the burden of terminal illness due to advanced, chronic disease is increasing in Indonesia, in the context of limited palliative and end-of-life care resource [12, 13]. Furthermore, research regarding end-of-life care in Indonesia is scarce. One factor which may hinder this and which has been observed in many contexts internationally, is a belief among Indonesian people that talking about death and dying is taboo and the assumption that such conversations may cause anxiety or depression [14].

### Objectives

This is the first study in Indonesia to describe lay and medical perspectives regarding the initiation of conversations about death and dying in the community setting, testing the hypothesis that most people would prefer to have end-of-life care discussions with a healthcare professional in the early stages of disease progression.

## Methods

### Study design

**Fig. 1:**
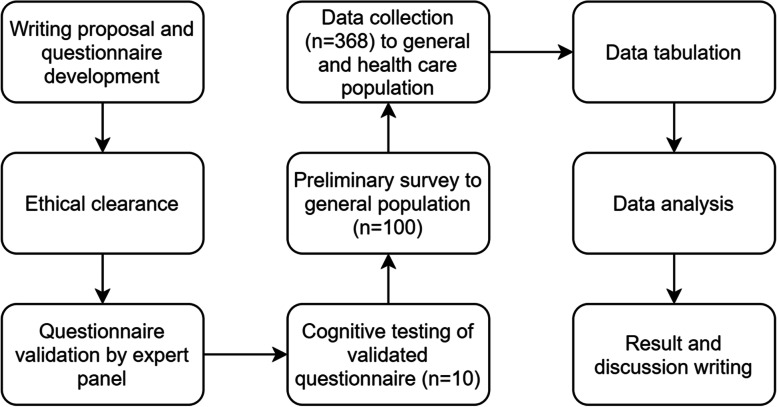
Overall study design

A cross-sectional descriptive approach was used to gain quantitative data regarding preferences relating to end-of-life care discussions in the Indonesian population. Figure 1 shows the overall study design.

### Setting

As this was the first study in Indonesia to focus on discussions about end-of-life care, a general population-based approach was taken in order to capture a baseline description of preferences in the community setting. A second group of respondents were recruited from the healthcare professional population to afford further insight into end-of-life care and provide a comparator group. Although the inclusion of a patient population may be considered to give a more realistic view of end-of-life care through authentic, lived experience, ethical considerations relating to this vulnerable group demand a greater understanding of the acceptance and possible harms of these conversations in Indonesia. Data was collected from March to November 2018.

### Instrument development and preliminary survey

In the absence of a validated end-of-life tool in Indonesia, a questionnaire in the national language (Bahasa Indonesia) was developed and validated in a preliminary study. This questionnaire was based on similar instruments used previously to study end-of-life preferences in Europe [15], USA [16], India [17], Japan [18]. Kenya [19] and Singapore [20]. (Questionnaire available in Bahasa Indonesia (Additional file 1a) and English versions (Additional file 1b)).

The questionnaire underwent face validity with a panel of 12 local experts in palliative care and communication (profiles detailed in Additional file 2). After two rounds of content and construct validation, using the Validation Rubric for Expert Panel (VREP)©, tool [21] (Additional file 3), the questionnaire underwent further cognitive testing with 10 participants. These participants were purposefully selected to represent the general population in terms of age (21–65 years), gender and educational background (lower secondary to bachelor’s degree). Subsequent to this, some terminologies were simplified to enhance understanding.

Feedback from cognitive testing indicated some resistance towards talking about end-of-life. A preliminary survey to understand this was therefore conducted in one hundred participants selected at random from the general population using a consecutive sampling method [22] via an third-party, open survey platform [23].The majority of respondents to this survey (75%) felt comfortable discussing end-of-life issues.

### Questionnaire distribution

The questionnaire was distributed to the general population using the consecutive sampling method via the online survey platform *Jakpat* [23]. Jakpat is an Indonesian Open Survey platform with 611,000 mobile, in-country respondents and has conducted of 10,000 online surveys for various companies, including the Indonesian Institute of Sciences [23]. Participants were all over the age of 21, the age of consent in Indonesia. Internet penetration rates in Indonesia is considerably higher in younger age groups compared with older people [24]. Therefore, to mitigate falsely skewed results and better reflect national age distribution, a special sampling criterion was included to ensure 20–30% of respondents were above 40 years of age. A target of 200 respondents was set as an adequate sample size for this initial descriptive study, with time limit of 2 weeks to gather data.

The same questionnaire was distributed to healthcare professionals using the snowball sampling method via a WhatsApp group of professional networks and with a target of 100 responses. Members of this group were invited to forward the questionnaire to other healthcare professionals only.

### Data analysis

Statistical analysis used IBM SPSS Statistic tools v.20. Univariate analysis was used to obtain descriptive data to obtain median and mean of numeric data. Normality test of data distribution was performed using coefficient of variance and Kolmogorov-Smirnov tests. Bivariate analysis using chi square test and Fisher exact test was used for comparison of the general and healthcare professional population results. To control for population characteristics due to different sampling methods between the two groups, multivariate analysis using binary logistic regression was performed in comparing decisions to discuss diagnosis and life expectancy.

### Ethical issues

Approval from the medical ethics committee of Newcastle University was granted prior to study commencement. All participants in the research were healthy, adult volunteers and were presented beforehand with written explanations of the research, questionnaire, anonymity and data confidentiality. Participants were able to leave the study at any time and required to confirm informed consent prior to completing the survey. There is no conflict of interest to disclose.

## Results

### Respondent demographics

A total of 380 participants responded to the questionnaire (266 general population; 114 healthcare professionals). After removal of incomplete data, results from 368 participants (255 general population; 113 healthcare professionals) were analysed.

Respondent demographics are shown in Table 1. Overall, 22 of Indonesia’s 34 provinces were represented. The average age of respondents was 32 years for the general population and 29 years for healthcare professionals, compared with the Indonesian national average of 30 years [9]. The percentage of respondents by religion similarly accords with national data, with Islam as the majority (78% compared with Indonesian figure of 87%) [25]

**Table 1:** Respondent Demographics

Characteristics	General population (*n* = 255)	Health care provider (*n* = 113)	National Average [9, 25]
Age mean (range) (years)	32 (21–70)	29.27 (26–40)	30
40–59	88 (34.5%)	1 (0.9%)	
> =60	3 (1.2%)	0 (0%)	
Gender			
Female	121 (47.4%)	59 (52.2%)	50.2%
Male	134 (52.6%)	54 (47.8%)	49.8%
Education level			
Elementary	10 (3.9%)	0 (0%)	
Junior high school	13 (5.1%)	0 (0%)	
High school	103 (40.4%)	1 (0.9%)	62.0%
Diploma 1	5 (2.0%)	0 (0%)	
Diploma 2	1 (0.4%)	0 (0%)	
Diploma 3	26 (10.2%)	0 (0%)	
Bachelor/undergraduate	87 (34.1%)	97 (85.8%)	12.0%
Specialist/Post Graduate	10 (3.9%)	15 (13.3%)	
Religion			
Moslem	219 (85.9%)	69 (61.1%)	87.0%
Christian	28 (10.9%)	37 (32.7%)	
Hindu	3 (1.2%)	3 (2.7%)	
Buddhist	4 (1.6%)	4 (3.5%)	
Others	1 (0.4%)	0 (0%)	
Specialty			
General Practitioner		43 (38.0%)	
Resident		57 (50.4%)	
Specialist		9 (8.0%)	
Nurse		3 (2.7%)	
Other		1 (0.8%)	

The majority of respondents had fulfilled the national requirement of 12 years of basic education, up to and including level 2 (lower secondary) education. Level 3 (upper secondary) was the highest level of education attained by 62% of the Indonesian population, compared to 9% (23/255) of general population respondents in our study. Thirty eight percent of the general population group (97/255) had completed undergraduate education or higher (level 5 and above) compared to 99% of the healthcare professional group (112/113) and a national average of 12%. The healthcare professionals group comprised general practitioner, resident, dentist, nurse and medical specialists.

### Initiation of end-of-life care discussion

Result presented in Table 2. Overall, most respondents (80%) stated they would like a healthcare professional to discuss end-of-life care with them (296/ 368). This was more marked in the healthcare professionals group compared with the general population (94% v 75%, *p* < 0.001) (Table 2). Of those who responded positively to discussion, 63% (185/296) expressed a preference for this to happen at first diagnosis or when the patient asks for it (24%, 70/296). The preference for end-of-life discussion at diagnosis was significantly higher in the general population compared with health care professionals (68% vs 52%, *p* < 0.001).

**Table 2:** Respondents’ preference regarding end-of-life care discussion initiation and depth of information

Do you want healthcare provider to discuss about End-of-life Care?			
Yes	190 (74.5%)	106 (93.8%)	< 0.001*
If yes the discussion should start at^			
First diagnosis	130 (68.4%)	55 (51.9%)	0.017**
When patient ask for it	39 (20.5%)	31 (29.2%)	
Initiation of therapy	15 (7.9%)	12 (11.3%)	
Right before discharge	6 (3.2%)	7 (6.6%)	
Other	0 (0%)	1 (0.9%)	
If yes.who should initiate the discussion^			
Doctor	112 (58.9%)	76 (71.1%)	0.036**
Myself (by my request)	74 (38.9%)	26 (24.5%)	
Nurse	3 (1.6%)	2 (1.9%)	
Other	1 (0.5%)	2 (1.9%)	
In case of terminal illness.do you want to know about			
Diagnosis			
Yes	232 (91.0%)	112 (99.1%)	0.004*
Life expectancy			
Yes	186 (72.9%)	94 (83.2%)	0.034*
Who else do you wish to know above information?			
Spouse	184 (72.2%)	100 (88.5%)	0.001*
Parent	139 (54.5%)	62 (54.9%)	0.949*
Child	90 (35.3%)	58 (51.3%)	0.004*
Friend	38 (14.9%)	14 (12.4%)	0.523*
Other	1 (0.4%)	2 (1.8%)	0.224**
No One	43 (16.9%)	4 (3.5%)	< 0.001*

^ binary logistic regression after adjusted by age *chi-square ** fisher test

Answer categories presented as percentage for each group (column percentage). Statistical tests compare general population and healthcare professionals’responses, with significant difference value (*p* < 0.05) in bold

The preferred person to start the discussion was the doctor (overall 64%) with healthcare professionals favouring this preference compared to the general population (71% v 59%, *p* = 0.036). A third of all respondents stated that they would like to initiate the discussion (overall 34%; general population 39%; healthcare professionals 25%). Fewer than 2% of all respondents preferred a nurse to initiate discussions (overall 1.7%; general population 1.6%; healthcare professionals 1.9%).

### Diagnosis of end-of-life and life expectancy

Overall, 94% (344/368) wanted to be informed when they were in the terminal phase of their illness (general population 91%; healthcare professionals 99.1%) and 76% would like to know their life expectancy (general population 73%; healthcare professionals 83%).

When adjusted for age, healthcare professionals are more inclined to want to know their diagnosis and prognosis compared with the general population (99% v 91 and 84% v 73% respectively). Most participants overall would like their spouse or parent to know about their diagnosis (93%) and life expectancy (76%). Health care professionals were significantly more likely than the general population to include their spouse (89% v 72%, *p* = 0.001) and child (51% v 36%, *p* = 0.004) in these conversations. Conversely, more respondents from the general population would prefer no one else to know the information (17% v 4%, *p* < 0,001).

## Discussion

The majority (80%) of our study respondents would like a healthcare professional to discuss end-of-life care in case of terminal illness with them. This accords with previous calls for physicians to routinely initiate such discussions and provide time for palliative care patients and their families to discuss end-of-life care [26, 27]. Our finding that healthcare professionals were more likely to desire end-of-life care discussions for themselves compared to the general population may be due to accustomed readiness to accept medical information but also suggests an implicit acknowledgement of the importance of these conversations.

### Healthcare professional-related factors

Our results concur with Keating et al. [28], who investigated responses of physicians presented with a hypothetical situation of newly diagnosed metastatic cancer. In common with this study, most healthcare professionals expressed a preference to discuss prognosis primarily at the time of diagnosis (52% in our study; 65% in Keating et al [28]) and secondarily when initiated by the patient (29% in our study; 15% in Keating et al [28]). Similarly, Keating et al [28] found younger physicians more open to discussing end-of-life and initiating these conversations earlier in the disease trajectory compared with their older counterparts. The majority of healthcare professional in our study were early and mid-career physicians, with an average age of 29 years, which may contribute to their apparent eagerness towards initiating and discussing end-of-life issues. Whether senior physicians have similar preferences compared to their junior colleagues is open for further exploration.

It must be acknowledged that our findings relate to a situation abstracted from authentic practice. In real situations, the evidence is that physicians do not regularly initiate end-of-life discussions until late in the course of an illness. In their study of patients with metastatic lung cancer, Huskamp et al. [29] found that most had no discussion about end-of-life care within 4–7 months of diagnosis, despite poor 5-year survival rates of 5–24%. Another study among heart failure patients also noted that more than half of physicians feel hesitant to mention end-of-life care or perceived patients were not ready to talk about the issue [30]. Indeed, only 12% of physicians reported routinely discussing end-of-life with their heart failure patients, as advocated by the American Heart Association [31], suggesting that clinicians’ reluctance to discuss death and dying with patients is independent of diagnosis. The statistically significant difference in the general population’s preference for end-of-life discussion at diagnosis compared with healthcare professionals that we observed corroborates these studies. Interestingly, we demonstrated a seeming contradiction in that healthcare professionals were statistically more likely to prefer a doctor to initiate end-of-life discussions compared to the general population.

### Patient-related factors

The risk of a patient’s mental capacity deteriorating as their disease progresses and the potential benefits of planning care, setting goals, maintaining autonomy, managing expectations and increasing patient and carer satisfaction demonstrates the importance of conducting end-of-life discussions in a timely manner. Furthermore, avoiding futile treatments protects patients from harm, respects their dignity and saves resources to the wider healthcare economy [32]. Conversely, late discussion and late referral to hospice is associated with poorer patient quality of life and worse bereavement adjustment [32]. This study adds to the body of evidence recommending initiation of end-of-life discussions in the early course of disease, by demonstrating a preference for this within the general population of Indonesia. We accord with Wright et al. [33] that physician reluctance exceeds their patient’s readiness to discuss the topic early in their disease trajectory.

Age may affect an individual’s readiness to talk about death and dying, with several studies demonstrating various results in different countries. Ang et al [20] describe differences between age groups among Singaporeans regarding regarding end-of-life care preferences, research comprising mostly young adults and therefore similar to our study population. Studies in Taiwan and Iran have found significantly higher levels of fear and anxiety associated with death in the elderly compared with younger populations [34, 35]. In contrast, Chan and Yin showed that age did not affect the level of anxiety between people below and above 40 years of age in Malaysia [36], although this study was skewed towards younger age groups with only 3 participants aged over 60. Thus we suggest that age is a factor to be considered in further research regarding end-of-life discussions.

### Initiating discussions

Our study found the overwhelming majority of respondents from both the general population and healthcare provider groups preferred a doctor to initiate dialogue about end-of-life care. Davison [37] demonstrated that patients with end stage renal failure undergoing dialysis perceived physicians as the primary source of information and therefore responsible for initiating and guiding advance care planning. We did not, however, confirm Davison’s observation that some participants would accept a nurse or social worker to commence discussion [37], although she caveates that professionals other than doctors were acceptable contingent upon their involvement in the patient’s care and our questionnaire was posed hypothetically. A systematic review by Adams et al. [38] demonstrated strong evidence for the role of nurses in actively brokering decision-making at the end-of-life among family members and the healthcare team. Indeed, the American Nursing Association stresses the importance of nurses advocating for patients’ preferences, including at the end-of-life [39]. We therefore argue for further research to assess physician and family member’s perception of the nurse role in end-of-life discussions, and to better understand authentic experiences regarding nurse participation.

### Information sharing

Our study, concurring with Leydon et al. [40], found that although most people would like to know about diagnosis, fewer wanted information regarding prognosis. Furthermore, we observed a similar difference between preference for knowledge about diagnosis compared with prognosis in both study populations (15% difference in healthcare professionals; 18% in the general population).

According to Walczak [26], doctors are reluctant to discuss life expectancy for fear of destroying hope and causing death anxiety and may collude with patients to avoid these conversations. Krawczyk and Gallagher [41] suggest that by eliciting suspicion of false hope and using confusing euphemisms, prognostic uncertainty may harm the doctor-patient relationship, especially when there is a perceived incongruence between the doctor’s message and the aggressiveness of care provided. They also demonstrated through retrospective reports from bereaved relatives that effective communication of prognostic information helped them to prepare and satisfaction of care was higher [41]. We recommend further exploration regarding information needs and preferences, in order to improve professionals’ confidence in leading conversations about end-of-life care.

Our study found the majority of respondents would wish to know about their prognosis adding evidence to the recommendation that physicians initiate dialogue about end-of-life early in the disease trajectory, tailoring the amount of information to the patient concerned and as part of an ongoing plan of care involving patient and their family.

Most of our participants would like their family, particularly their children, to know about their diagnosis of terminal illness and life expectancy. This was an unexpected finding and further research into the role of offspring in discussions about the end of their parent’s life is warranted.

Overall, we observed positive acceptance towards end-of-life care discussion in terminal illness, refuting the negative assumption among Indonesian people regarding communication of death and dying [11]. Considering that reluctance to talk about end-of-life still exists among physicians, this study should give healthcare professionals confidence to initiate conversations about death and dying earlier in the course of disease to achieve better goals of care. The questionnaire used in this study is the first of its kind regarding end-of-life care tailored to the Indonesian cultural and language and may serve as basis for practice and policy developments in this field.

## Study limitation and suggestions

This study has a number of limitations, suggesting improvements for subsequent research. Firstly, although the number of respondents (*n* = 368) was adequate for statistical analysis, a larger sample with more varied respondent demographics would better reflect Indonesia’s vast and multicultural background. Secondly, although broadly representative of the Indonesian population, this study did not comprehensively explore the effect of age and mortality threat on preferences for end-of-life discussions because the majority of respondents in this study were young adults and likely in good health. Thirdly, respondents in both study populations have attained higher levels of education compared with the national average which may affect beliefs and understandings of health and wellbeing, and consequently influence preferences regarding end-of-life care. Further paper- or interview-based investigations to reach respondents with lower education attainment and lower digital literacy are warranted. It must also be noted that this research was undertaken in 2018, before the COVID-19 pandemic, whose impact on the Indonesian population’s attitudes and perceptions towards death and dying is as yet unknown. Therefore, we recommend repeating this study post-pandemic, suggesting this may yield different results.

## Conclusions

This study developed and validated an instrument to capture preferences regarding discussions about end-of-life care in Bahasa Indonesia, adjusted to local culture. Our findings counter the preconceived notion that talking about death and dying is taboo in the Indonesian culture. We believe this study makes an important contribution to the clinical and policy-driven agenda to improve palliative care services in developing countries such as Indonesia, by supporting healthcare professionals to discuss end-of-life care with their patients, respect autonomy and develop meaningful, appropriate and patient-centred services accordingly.

## Supplementary Information

**Additional file 1.**

**Additional file 2.**

**Additional file 3.**

## Data Availability

The datasets used and/or analysed during the current study are available from the corresponding author on reasonable request.

## Abbreviations

UK: United Kingdom
VREP: Validation Rubric for Expert Panel
IBM SPSS: International Business Machines Corporation Statistical Package for the Social Sciences
COVID-19: Coronavirus Disease of 2019
