# Supplementary material for: Preference for initiation of end-of-life care discussion in Indonesia: a quantitative study
Source: BMC Palliat Care. 2022 Jan 6;21:6. doi: 10.1186/s12904-021-00894-0 (PMC8733905; doi:10.1186/s12904-021-00894-0)
Supplement: Supplementary file 1 — Additional file 1. [file 12904_2021_894_MOESM1_ESM.zip › Additional File 1a (Questionnaire - Bahasa)R3.docx]

**Additional File 1a (Questionnaire - Bahasa)**

**PILIHAN PERAWATAN AKHIR KEHIDUPAN**

Terima kasih banyak atas kesediaan Anda menyediakan waktu untuk proyek ini. Studi ini bertujuan memahami pandangan dan pilihan masyarakat Indonesia tentang perawatan akhir kehidupan (*end of life care*).

**Apa yang dimaksud perawatan akhir kehidupan?**

Perawatan yang diberikan terutama pada pasien dengan penyakit berat atau terminal (penyakit dimana diperkirakan kematian dapat terjadi dalam waktu 6 bulan atau kurang). Tujuan perawatan adalah menangani nyeri, keluhan fisik dan psikologis, serta meringankan penderitaan demi kualitas hidup yang baik hingga kematian.

**Mengapa diperlukan pemahaman yang baik tentang pilhan perawatan akhir kehidupan?**

Selama memungkinkan, setiap orang dapat memiliki pilihan pribadi tentang perawatan dirinya sendiri hingga akhir kehidupan dan kematian. Misalnya pilihan tempat perawatan, pendamping/perawat, jumlah informasi, dan alat bantu medis yang dipakai. Sayangnya seringkali hal tersebut tidak dibicarakan atau dicatat dengan baik, sehingga kualitas pelayanan kurang memuaskan.

Apabila Anda bersedia ikut serta, silakan menandatangani lembar persetujuan dan menjawab pertanyaan berikut.  Anda bebas memilih untuk melewati atau tidak menjawab pertanyaan yang membuat Anda merasa tidak nyaman, atau pun membatalkan partisipasi.

Kerahasiaan data Anda juga dijamin dan hanya akan dilihat oleh tim peneliti serta penyebaran hasil hanya dilakukan secara ilmiah tanpa mengungkapkan identitas peserta secara pribadi.

Data peneliti:

Venita Eng

Venita.w@gmail.com

Dokter Pelayanan Sosial Yayasan Kanker Indonesia DKI Jakarta/

Mahasiswa Program Pascasarjana Newcastle University, UK

**LEMBAR PERSETUJUAN**

Saya yang bertanda tangan di bawah ini, telah menerima informasi yang benar dan jelas mengenai latar belakang, tujuan, dan metode penelitian yang berjudul "Investigasi Preferensi Perawatan Akhir Kehidupan pada Penduduk Indonesia ".

Saya mengerti bahwa informasi yang saya berikan akan bermanfaat untuk meningkatkan kualitas perawatan kesehatan di Indonesia. Saya juga mengerti bahwa setiap informasi yang saya berikan akan tetap anonim dan rahasia dan tidak akan dibagi ke pihak ketiga di luar tim peneliti. Saya mengizinkan diseminasi hasil penelitian ini di forum ilmiah atau publikasi selama tetap terjaga kerahasiaannya secara personal.

Tanggal dan waktu,

(tanda tangan)

(nama jelas)

**Subtopik Kuesioner**

Karakteristik responden (silakan memberikan tanda centang/melingkari satu poin yang sesuai dengan kondisi anda saat ini) :

1. Usia:
2. Total pendapatan / bulan :
   1. Kurang dari Rp 3.700.000
   2. Rp 3.700.001 - Rp 10.000.000
   3. Rp 10.000.001 - Rp 40.000.000
   4. Rp 40.000,001 - Rp 100.000.000
   5. Lebih dari Rp 100.000.000
3. Latar belakang pendidikan :
   1. Tidak tamat SD
   2. SD
   3. SMP
   4. SMA
   5. Perguruan tinggi
4. Jumlah anak :
5. Kota tempat tinggal saat ini :
6. Agama:

**Pertanyaan (silahkan memberi tanda centang/melingkari poin yang sesuai dengan pilihan anda)**

1. **Awal diskusi perawatan akhir hidup**

a)       Jika Anda menderita penyakit berat atau terminal, apakah Anda ingin tenaga medis membicarakan tentang perawatan akhir kehidupan ?

- Ya
- Tidak

b)      Jika ya, kapan menurut Anda pembicaraan tentang perawatan akhir kehidupan sebaiknya dimulai?

- pada saat pertama kali dinyatakan menderita penyakit berat/terminal
- pada saat terapi di Rumah Sakit dimulai
- pada saat pasien akan pulang ke rumah
- saat pasien menyatakan ingin mendiskusikan hal tersebut
- lainnya................................................. .....................

c)        Menurut Anda siapa yang sebaiknya memulai pembicaraan perawatan akhir kehidupan?

- Dokter
- Perawat
- Diri sendiri (permintaan sendiri)
- Lainnya (sebutkan.............................................. ...

d)       Jika suatu saat Anda menderita penyakit terminal, apakah Anda ingin tenaga kesehatan memberitahu nama penyakit Anda?

- Ya
- tidak

e)        Jika suatu hari Anda menderita penyakit terminal, apakah Anda ingin tahu tentang berapa lama perkiraan waktu hidup Anda ?

- Ya
- Tidak

f)       Siapa saja yang Anda inginkan untuk ikut mengetahui informasi di atas?

- Pasangan
- Anak-anak
- Teman
- Lainnya (sebutkan......................
- tidak seorangpun

**DEBRIEF**

Terima kasih banyak atas waktu dan kesediaan Anda untuk berpartisipasi dalam penelitian ini

Sekali lagi, saya menjamin Anda bahwa informasi yang Anda berikan akan dijaga kerahasiaannya dan hanya akan dipergunakan untuk tujuan ilmiah serta upaya peningkatan kualitas pelayanan kesehatan.

Apabila Anda memiliki pertanyaan atau masukan lebih lanjut, mohon jangan ragu untuk menghubungi peneliti
