# Supplementary material for: Preference for initiation of end-of-life care discussion in Indonesia: a quantitative study
Source: BMC Palliat Care. 2022 Jan 6;21:6. doi: 10.1186/s12904-021-00894-0 (PMC8733905; doi:10.1186/s12904-021-00894-0)
Supplement: Supplementary file 1 — Additional file 1. [file 12904_2021_894_MOESM1_ESM.zip › Additional file 1b (Questionnaire-English)R3.docx]

**Additional file 1b (Questionnaire-English Version)**

**END OF LIFE CARE PREFERENCES**

Thank you very much for your willingness to make time for this project. The aim of this study is to understand the view and preferences of Indonesian people regarding end of life care *(end of life care).*

**What is an end of life care?**

A care given especially to patients with severe or terminal disease (a disease where it is estimated that death can occur within 6 months or less). The goal of treatment is to manage pain, physical and psychological discomfort,  and relieve suffering for good quality of life and quality of death .

**Why is it necessary to have a** **good** **understanding** **of end of life** **care?**

For as long as possible, everybody should have the chance to choose what kind of care they receive until the end of life. For example the choice of place of care, companion / caregiver, amount of information, and medical aids used. Unfortunately it is often not discussed or documented properly, thus  the quality of care is less satisfactory.

If you are willing to participate, please sign the consent form and answer the following questions. You are free to skip a question that makes you feel uncomfortable, or to cancel participation.

The confidentiality of your data is also guaranteed and will only be seen by the research team as well as the dissemination of results is only done scientifically without disclosing the identity of the participants personally.

Research data:

Venita Eng

Venita.w@gmail.com

Doctor Social Services Yayasan Kanker Indonesia DKI Jakarta /

Student Postgraduate Program Newcastle University, UK 

**APPROVAL SHEETS**

I, the undersigned, have received accurate and clear information about the background, objectives, and methods of research entitled "Investigation of End Care Preferences   Life on Indonesian Population   ".

I understand that the information I provide will be useful for improving the quality of health care in Indonesia.   I also understand that any information I provide will remain anonymous and confidential and will not be shared with third parties outside   team   researchers .   I allow the dissemination of research results at a scientific forum or publication during its confidentiality is maintained personally.

Date and time,

(signature)

(clear name)

**Questionnaire Subtopic**

Characteristics of respondents (please tick / circle one point according to your current condition) :

a)       Age :

b)       Total income   / month   :

1. Less than Rp 3.700.000
2. Rp 3,700.001 - Rp 10.000.000
3. Rp 10.000.001 - Rp 4 0.000.000
4. Rp 4 0.000,001 - Rp 100,000,000
5. Over Rp 100,000,000

c)        Educational background :

1. Not completed primary school
2. SD
3. SMP
4. SMA
5. College

e)       Number of children :

h)       Religion:

**Questions (please tick / circle the points corresponding to** **your** **choice** **)**

**Initiation of End-of-life care discussion**

a )         If you have a severe or  terminal illness,   Do you want your healthcare provider  to discuss about end-of-life care ?

- Yes
- No

b)        If yes, When do you think the discussion about End-of-Life care should be started?

- at the first time it was  stated as severe/terminal illness
- when the therapy in hospital begin
- when patient about to go home
- when patient said he/she wants to discuss about it
- other ................................................ . .....................

c)          Who do you think should initiate discussion about end-of-life care?

- Doctor
- Nurse
- Yourself (by own request)
- Others  ............................................... ..

d)         If one day you suffer from terminal illness, do you want your healthcare provider to tell you the name of your disease?

- Yes
- no

e)          If one day you suffer from terminal illness, would you like to know how long is your life expectancy?

- Yes
- No

f)         Who else do you wish to know the above informations?

- Spouse
- Children
- Parents
- Friend
- Others (specify ......................

**DEBRIEF**

Thank you very much for your time and willingness to participate in this research

Again, I assure you that the information you provide will be kept confidential and only it will be used for scientific purposes as well as improving the quality of health services.

If you have further questions or feedback, please do not hesitate to contact the researcher.
