# Supplementary material for: Preference for initiation of end-of-life care discussion in Indonesia: a quantitative study
Source: BMC Palliat Care. 2022 Jan 6;21:6. doi: 10.1186/s12904-021-00894-0 (PMC8733905; doi:10.1186/s12904-021-00894-0)
Supplement: Supplementary file 2 — Additional file 2. [file 12904_2021_894_MOESM2_ESM.docx]

**Additional File 2 - Expert Panel Profile**

The round of experts to evaluate content and construct validity consists of:

1. Prof.Dr.dr.Aru Wisaksono Sudoyo, Ph.D, SpPD-KHOM  : a renowned hematologist-oncologist in Indonesia, also a Professor in Medical Faculty and National Central General Hospital in Indonesia (known as RSCM, the highest referral center for healthcare in Indonesia), with over than 30 years of experience in care for cancer patient and providing palliative care as well.
2. Dr.Maria A.Witjaksono, MpallC, PC Physician : a renowned expert in palliative care in Indonesia, formerly head of palliative care unit in Central Cancer Hospital in Indonesia, with over than 20 years of experience in palliative and end-of-life care.
3. Dr.Rudi Putranto,Sp.PD-Kpsi : an internist with specialty in psychosomatic disease, he is currently the head of palliative care unit and program in National Central General Hospital in Indonesia
4. Dr.Hilda Angrianita,MPH : physician with degree in public health,she is currently in charge as palliative case manager in national central general hospital and direct care for patient.
5. Dr.Diah Martina,SpPD : an internist currently registered in PhD of palliative care program in Netherland, she has conducted several studies and write papers and articles regarding palliative care in Indonesia, she also has experience as medical journalist in several medical and popular publication.
6. Dr.Endang Windiastuti,Sp.A(K) : consultant in pediatric oncology with over than 30 years of experience in oncology and palliative care.
7. Tika Olimvia,S.Ikom : holding a degree for communication, she has been actively involved in cancer organization for 5 years and deal with patient advocacy as well.
8. Aryanthi Baramuli Putri : founder of CISC (cancer information and support center), a group of 151 cancer survivors from all over of Indonesia. She is a breast cancer survivor and actively advocate for patient right and access of healthcare. She provides input from survivor and patient point of view.
9. Dr.Rebecca N.Angka,Mbiomed : head of outpatient cancer clinic with experience of mentoring palliative care training
10. Veronica Triyanti, Psi. : holding a master degree in psychology and over than 10 years of practice experience in Indonesia
11. Roslina Sinaga,S.Pd : 35 years of experience as school teacher, headmistress, and official at ministry of culture and education.
12. Susi Susilawati, S.Kep,Ns. : a palliative care nurse with 9 years’ experience of patient care and lecturer in pediatric palliative care.
