# Supplementary material for: Preference for initiation of end-of-life care discussion in Indonesia: a quantitative study
Source: BMC Palliat Care. 2022 Jan 6;21:6. doi: 10.1186/s12904-021-00894-0 (PMC8733905; doi:10.1186/s12904-021-00894-0)
Supplement: Supplementary file 3 — Additional file 3. [file 12904_2021_894_MOESM3_ESM.docx]

**Additional File ppendix 3 – VREP© Validation Form by Marilyn K. Simon (***with permission of author***)**

**Survey/Interview Validation Rubric for Expert Panel - VREP**©

| **Criteria** | **Operational Definitions** | **Score**  **1=Not Acceptable** (major modifications needed)  **2=Below Expectations** (some modifications needed)  **3=Meets Expectations** (no modifications needed but could be improved with minor changes)  **4=Exceeds Expectations** (no modifications needed) | | | | **Questions NOT meeting standard**  **(List page and question number) and need to be revised.**  ***Please use the comments and suggestions section to recommend revisions.*** |
| --- | --- | --- | --- | --- | --- | --- |
|  |  | 1 | 2 | 3 | 4 |  |
| **Clarity** | - The questions are direct and specific. - Only one question is asked at a time. - The participants can understand what is being asked. - There are no *double-barreled* questions (two questions in one). |  |  |  |  |  |
| **Wordiness** | - Questions are concise. - There are no unnecessary words |  |  |  |  |  |
| **Negative Wording** | - Questions are asked using the affirmative (e.g., Instead of asking, “Which methods are not used?”, the researcher asks, “Which methods *are* used?”) |  |  |  |  |  |
| **Overlapping Responses** | - No response covers more than one choice. - All possibilities are considered. - There are no ambiguous questions. |  |  |  |  |  |
| **Balance** | - The questions are unbiased and do not lead the participants to a response. The questions are asked using a neutral tone. |  |  |  |  |  |
| **Use of Jargon** | - The terms used are understandable by the target population. - There are no clichés or hyperbole in the wording of the questions. |  |  |  |  |  |
| **Appropriateness of Responses Listed** | - The choices listed allow participants to respond appropriately. - The responses apply to all situations or offer a way for those to respond with unique situations. |  |  |  |  |  |
| **Use of Technical Language** | - The use of technical language is minimal and appropriate. - All acronyms are defined. |  |  |  |  |  |
| **Application to Praxis** | - The questions asked relate to the daily practices or expertise of the potential participants. |  |  |  |  |  |
| **Relationship to Problem** | - The questions are sufficient to resolve the problem in the study - The questions are sufficient to answer the research questions. - The questions are sufficient to obtain the purpose of the study. |  |  |  |  |  |
| **Measure of Construct:**  **A: (        )** | - The survey adequately measures **preferred place of end-of-life care** |  |  |  |  |  |
| **Measure of Construct:**  **B:  (        )** | - The survey adequately measures **initiation of end-of-life care discussion** |  |  |  |  |  |
| **Measure of Construct:**  **C: (        )** | - The survey adequately measures **dying/death preferences** |  |  |  |  |  |
| **Measure of Construct:**  **D:  (        )** | - The survey adequately measures **end-of-life care life support provision** |  |  |  |  |  |
| **Measure of Construct:**  **D:  (        )** | - The survey adequately measures **willingness for end-of-life care discussion** |  |  |  |  |  |

*Permission to use this survey, and include in the dissertation manuscript was granted by the author, Marilyn K. Simon, and Jacquelyn White.  All rights are reserved by the authors. Any other use or reproduction of this material is prohibited.*

By Marilyn K. Simon with input from Jacquelyn White<http://dissertationrecipes.com/>
